# Iranian’s healthcare system challenges during natural disasters: the qualitative case study of Kermanshah earthquake

**DOI:** 10.1186/s12873-020-00359-2

**Published:** 2020-09-24

**Authors:** Mohammadtaghi Mohammadpour, Omid Sadeghkhani, Peivand Bastani, Ramin Ravangard, Rita Rezaee

**Affiliations:** 1 Student Research Committee, Shiraz University of Medical Science, Shiraz, Iran; 2 Health Human Resources Research Center, School of Management and Medical Informatics, Shiraz University of Medical Science, 29 Ghasrodasht Street, Shiraz, Iran

**Keywords:** Natural disasters, Kermanshah, Healthcare system, Crisis

## Abstract

**Background:**

In order to the significance of lessons learned from the natural disasters for health care systems particularly in developing and under-developed countries, the main purpose of this study was to identify challenges and limitations in light of the earthquake experience in Kermanshah Province.

**Methods:**

The present study was conducted in 2019 as a qualitative research using content analysis method. In this regard, 19 key informants were selected using snowball sampling. To enhance the accuracy of the study, the four validation criteria for qualitative studies in data coding developed by Guba and Lincoln including credibility, transferability, dependability and confirmability were used. Data was analyzed applying Graneheim and Lundman (2004) approach for analyzing the qualitative content of an interview text.

**Results:**

Analysis of the data led to the identification of 2 main themes, 5 sub- themes and 17 main categories. The first main theme was health system oriented challenges containing challenges of medication supply and preparation, structural challenges, challenges in crisis-scene management and challenges of service delivery and the second main theme was non-health system oriented challenges including social and psychosocial challenges.

**Conclusion:**

According to the results, along with health system oriented challenges with the inter-sectoral or intra-sectoral nature, the non-health system oriented challenges the same as social, cultural and psychological factors can be considered as the major challenges of Iran’s healthcare system in the face of crises. This complicated context can shed the light to policy makers that not only attention to the medicine and medical equipment supply chain, manpower preparation and service delivery system can be considered as an emergency, but also careful attention to the structural challenges and crisis-scene management should be planned and considered as a priority. Besides, the policy makers and the local managers should try to plan and act in a contingent situation according to the social and cultural characteristics of the region and the psychological condition and the mental needs of the people.

## Background

All through history, human life and property has been continuously confronted with unpredicted events and sometimes frightening and dangerous disasters such as earthquakes, floods, storms, and wars [1]. Such accidents and catastrophes disrupt normal functioning in societies and also have a significant impact on people, activities, and their surrounding environments. They may be also caused by natural events (e.g. hurricanes, earthquakes, etc.) or arise out of human activities (namely, war, terror, biological and chemical warfare, etc.) [2]. According to the Center for Research on the Epidemiology of Disasters (CRED) although the number of disasters has actually plateaued over the last 20 years; this issue is yet considered important for many countries [3]. For instance, according to the results of the CRED only in 2018, 315 climate-related and geophysical disaster events were recorded all over the world [3]. Similarly, CRED have reported that at least 396 natural disasters have happened that led to 11,755 cases of killing people, affecting 95 million others and costing nearly 130 billion US$ [4]. Expecting a fivefold increase in natural disasters in the next fifty years [5, 6], millions of people are risking their lives due to damages and losses instigated by such events [7]. Even though only 1% of the world’s population is living in Iran, 6% of casualties resulted from natural disasters take place in this country [8], as one of the 10 most vulnerable nations across the world [8–10].

Evidence in this respect shows that healthcare systems need to have the distinguishing features of flexibility, strategic resource allocation, as well as strengthened health structures to deal with crises and natural disasters, considering the fact that the quality of such accidents demands flexibility, preparedness, and planning in advance because of its unpredictability [11]. The healthcare sector has thus a special place among all the elements involved in management of accidents and disasters, since the first and the most important public demands and concerns are associated with healthcare services. Preparedness is among the elements of crisis management and it simply requires planning, staff training, public education, practice, and assessment. At the individual level, this kind of preparation necessitates broadening knowledge along with improving attitudes and essential skills. At the local level, it stresses on developing programs, providing resources, and determining local management structure; and at the national level, it dictates setting policies, instructions, and practical guidelines. Unprepared healthcare systems dealing with crises and natural disasters can accordingly initiate catastrophic consequences as those observed in the 2010 Haiti earthquake or the floods in Pakistan [12]. Here, preparedness refers to coordinating all healthcare elements and sectors as grouped by the World Health Organization (WHO), including service delivery, health personnel, health information, medical technologies, financing, and trusteeship [13]. In addition to the importance of comprehensive coordination of all the above-mentioned cases, healthcare systems must put much emphasis on three key points of staff, infrastructure, and coordination in human resources sector to appropriately act in response and also pay special attention to first-line personnel providing services [14]. In Iran’s healthcare system, it is health workers’ responsibility to provide first-line services, so their crisis management skills and their training should be highlighted more than ever.

An efficient healthcare system can take action properly in critical situations with respect to complexities of patients’ medical conditions during crises; however, it can be really difficult to do planning and manage due to staffing and infrastructure constraints as well as extents of such events [15].

On November 25, 2018, Kermanshah Province in western Iran was hit by a 7.3 magnitude earthquake, and about 700 people lost their lives and more than 10 thousand were injured [16]. The main purpose of this study was to identify challenges and limitations of healthcare system in light of the earthquake experience in Kermanshah Province.

## Methods

The present study was conducted in 2019 as a qualitative research using content analysis method. In this study, semi-structured interviews aimed at explaining challenges facing Iran’s healthcare system in times of natural disasters were performed with senior members of Kermanshah Province Crisis Committee, emergency wards, Medical Supply Units, Food and Drug Administration executives of Red Crescent Society, as well as hospital heads involved in crisis, and financial managers. The interviewees were selected using snowball sampling method. Accordingly, the head of the Red Crescent Society and his deputy managers were first interviewed, and then they were requested to identify people who were experts in this field. The study participants were selected out of individuals well-informed about the crisis and how to cope with it, and also willing to share their information. At this stage, the interviews were done in-person at participants’ workplace according to their opinions and prior arrangements. The interviews were made by one of the researchers (OS). At the beginning of the interviews, general explanations were given about the study and its objectives and confidentiality of information was orally ensured. Moreover, all the individuals signed written consent forms and they were allowed to withdraw from the study at any stage of the study. The interviews lasted at least 50 min and they were conducted by one of the researchers. The given interviews were also recorded upon obtaining participants’ consent and transcribed verbatim just after they were completed. They also continued until saturation, after 19 interviews. To prepare the semi-structured interview guide consisting of 18 items, the related literature was reviewed and it was approved by two experts at Red Crescent Society. Moreover, face validity of the interview guide was assessed through conducting three initial interviews with the participants and some minor changes were done for better understanding of the questions. The content of these three interviews did not include in the final analysis.

To enhance the accuracy of the study, the four validation criteria for qualitative studies in data coding developed by Guba and Lincoln including credibility, transferability, dependability, and confirmability were used [17]. In order to enhance the credibility of the study, long-term involvement and continuous observations were exercised, so the researcher got fully involved in the study, properly established communications with participants, and accepted some deep concepts arising from the study. Moreover, integration of interviews and reviews of scientific texts was employed for this purpose. To increase the confirmability of the results, the encoded data were given to the participants to verify the accuracy of the results extracted. The conditions of the informants and the interview methods were clearly identified to augment the transferability of the study results. There were also attempts to select the study samples completely based on the objectives of the study and without any bias. Data analysis was also performed simultaneously with data collection to help the researcher become fully informed of the principles of the research. In order to enhance the dependability of the study results, methods utilized for coding concepts and themes, as well as textual and audio data became available. Furthermore, two members of the research team analyzed the content individually and discussed to reach agreement on their conflicts in order to ensure dependability.

The suggested approach of Graneheim and Lundman (2004) for analyzing the qualitative content [18] of an interview text was used. In this regard, the unit of analysis in this article was the interview texts about the challenges of healthcare system in light of the earthquake experience in Kermanshah Province. The meaning unit were extracted from the interview texts. Then these meaningful units became more concise and compressed to achieve to the condensed meaning unit. The codes then were extracted from these condensed meaning units and were labeled. The manifest analysis was used for extracting meaningful unites, condensed meaning units and the codes. After finalizing all the interviews` codes, the codes were integrated and categorized to appear the main category, sub-themes and finally by merging the sub-themes, the main themes were created. In this regard we try to use both manifest and latent analysis. Coding and categorization of the data were fulfilled manually instead of use of software in view of the fact that the transcribed texts of the interviews were in Persian and there was a need for increasing creativity. For this purpose, at the end of this step, challenges facing Iran’s healthcare system in times of natural disasters were identified and the results are tabulated and presented as a figure as well.

## Results

**Fig. 1:**
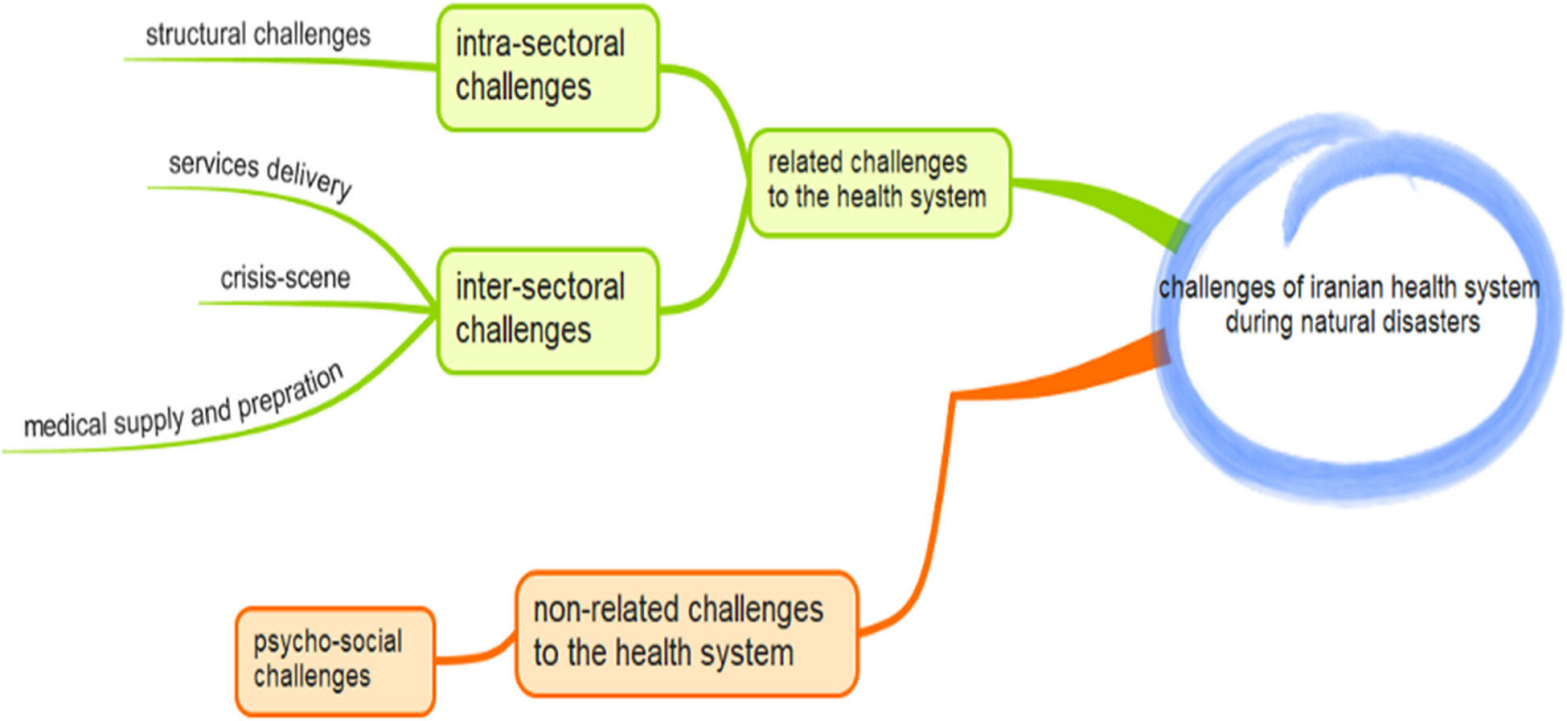
The map of the challenges in Iranian health system challenges during natural disasters

Manifest analysis of the data led to the identification of 62 final codes (Appendix). After continuing the analysis process via both manifest and latent analyzing, 17 categories were appeared that were merged and synthesized into 5 sub-themes and 2 main themes (Table1). According to Table 1, challenges of medication supply and preparation, structural challenges, challenges in crisis-scene management and challenges of service delivery are explored as the main health system oriented challenges and psychosocial and social challenges were among the non- health oriented challenges in during natural disaster in Iran. Figure 1 is illustrated the relationships among the main and the sub-themes and the main categories as well.

**Table 1:** Iranian’s Health System Challenges During Natural Disasters

Main themes	Sub themes	Main categories
**Health system-oriented challenges**	**Challenges of Medication Supply and Preparation**	Estimation of reasonable needs
		Strategic drug storage
		Supply-chain maintenance and promotion
		Domestic production
	**Structural Challenges**	Crisis management structure
		Coordination with stakeholders
		Planning in disasters
	**Challenges in Crisis-Scene Management**	Crisis financing
		Medical equipment
		Human resources
		Information management
	**Challenges of Service Delivery**	Medical infrastructure
		Pre-hospital emergency
		Healthcare services during crisis
**Non health system-oriented challenges**	**Psychosocial Social Challenges**	Trust-building
		Cultural conditions of the region
		Psychosocial and social interventions

### Health system-oriented challenges

The first theme recognized is health system oriented challenges. These kind of challenges are directly and without intermediators related to the health system. Although the nature of these factors are the same, some of them have the inter-sectoral and some have the intra-sectoral relationships. According to this some clarification of each of these sub-themes are as follows.

#### Challenges of medication supply and preparation

One of the most important challenges of the healthcare system in times of earthquake was associated with supplying and preparing medications. The main categories in this domain were estimation of reasonable needs, strategic drug storage, supply-chain maintenance and promotion, and domestic production. In this regard, one of the study participants had stated that:

“*It is necessary to make predictions before the crisis for medications, equipment, and supplies in different regions for specific periods in terms of population, size of the region, as well as geographical extent influenced by a crisis*” (P_10_).

With reference to the strategic drug storage, a participant had also said that:

“*In connection with single-use items such as stents, balloons, IUDs, and pacemakers as strategic commodities, we are totally dependent on other countries. So, we have stored them before crises and I think this can greatly help us.*” (P_9_).

Maintaining and promoting supply chain was correspondingly one of the key points deliberated. In this respect, one of the interviewees had reiterated that:

“*If the representatives of the largest medical supply companies were not accessible, we would face lots of troubles distributing resources in the region. Different companies at the provincial level helped a lot during recent earthquakes*” (P_13_).

#### Structural challenges

Another challenge identified in this study was structural challenges with the main categories of crisis management structure, planning, and coordination with stakeholders. Accordingly, the participants had accentuated the significance of defining the structure of crisis in healthcare system as well as identifying the role of governmental and non-governmental organizations in delivering services in times of crisis. In this regard, one of the participants had stated that:

“*If there was a systematic management headquarter dividing work, defining crisis management structure, and assuming responsibilities for crisis management and its subordinates such as rescue, healthcare management, accommodation, relocation, feeding, and crisis management as a whole, everyone could do their jobs properly*” (P_10_).

#### Challenges in crisis-scene management

Reflecting on challenges of crisis-scene management, the main categories of crisis financing, human resources, medical equipment, and information management were identified. In this respect, the study participants had assumed that:

“*On the subject of medical consumables required at the night when the earthquake struck the region, we sent some items just an hour later with the contribution of some companies*” (P_14_).

Or, elsewhere, an interviewee had added that:

“*We always give emphasis to human safety first and then work, but there was all the safety left. I went there in the early hours. There were many hospital personnel working with no gloves and they were operating and stitching under inappropriate conditions. They were also working in the sun and covered with dust*” (P_17_).

#### Challenges of service delivery

One other challenge in times of crisis was service delivery to the injured. Unpredictability of crisis could also make the given process challenging. The main categories of medical infrastructure, healthcare services during crisis, and pre-hospital emergency were thus ascertained. The participants’ opinions were also as follows:

“*… the only hospital in the city had been completely demolished and all the patients were merely being examined in the middle of the yard. There was no place where the medical staff could offer healthcare services. On the other hand, we were obliged to follow some standards for setting up a field hospital, that should be located in a known and accessible place to the public, so we launched a mobile medical system and a field hospital*” (P_6_).

Somewhere else, another interviewee had said that:

“*Our main problem was that we did not have helicopters with night-vision equipment. At the same time as the earthquake happened at 9 o’clock, we only had land transport that night but no air transport*” (P_3_).

Or, another participant had reiterated that:

“*Health packs are very helpful in times of crises, but disasters are characterized by different conditions and extents that can have effects on the design of such kits. We thus prepared basic health items including toothpastes and toothbrushes, towels, sanitary napkins, as well as mother-child packs, which could be of big help*” (P_2_).

### Non- health system-oriented challenges

The second theme explored in the present study, was the non-health system oriented challenges. These kind of challenges have the general nature depend on the social and cultural characteristics of the region and also the psychological condition after disaster. Although these challenges are not driven from the health system directly; the performance of the health system can affect them a lot and ignoring them can lead to great challenges along with those directly related to the health system.

#### Psychosocial-social challenges

Psychosocial- Social crises among the injured and their families were one of the challenges making crisis management difficult in this natural disaster in this study. This challenge was identified with the main categories of trust-building, cultural conditions of the region, and psychosocial interventions. Accordingly, the participants raised the need for a community mental health team in the region to assess the situation in coordination with other units affiliated to the Ministry of Health and Medical Education as well as other relevant organizations to design and implement appropriate interventions, aimed at promoting public mental health and preventing mental disorders caused by disasters. For example, the study participants had stated that:

“*In times of crisis, we do not usually confront with severe mental problems, but they generally occur after it. So, we recruited a psychologist in our visits and we also practiced mental health screening*” (P_16_).

And, elsewhere, one of the interviewees had said that:

“*Perhaps, we and the organizations that were in charge of helping the injured in previous crises could not do well, so we lost public trust*” (P_18_).

And, finally one of the interviewees had noted that:

“*People used to trust in governmental agencies easily, but unfortunately they lost it in the earthquake striking Sarpol-e Zahab County. It may be due to the performance of the organizations or even gossips and news about what had happened in previous crises*” (P_5_).

## Discussion

This study was aimed at exploring the Iranian health system challenges during disasters. Applying the four main criteria of Guba and Lincoln [17] that was presented in the method section for assuring the credibility, transferability, dependability and confirmability of the results, the analysis was implemented and the map of challenges were synthesized. Regarding the map, the challenges can be categorized in two main themes of health system oriented and non-health system oriented challenges. The health system related challenges may have an inter-sectoral or the intra-sectoral nature.

According to the present results the challenges related to service delivery, crisis scene management and medical supply and preparation are among the inter-sectoral challenges related to the health system.

Among challenges of medication supply and preparation, maintaining and promoting supply chain as well as improving performance especially in times of crisis are of utmost importance. Similarly, evidence suggests that supply-chain maintenance and promotion is in a highly weak position as crises and disasters occur [19]. One of the solutions advocated to maintain and improve supply chain during crises is increasing flexibility in collaboration with stakeholders and resources. This can immediately replace temporary resources and prevent drug shortages that is presented as a main category of medication supply and preparation. Some drug shortages, including anesthetics, were correspondingly identified among the challenges in this study. Furthermore, experience with natural disasters such as earthquakes in other countries demonstrates that one of the urgent needs is for emergency surgeries and surgical facilities such as operating rooms and anesthetics, but providing these mediations as strategic items in times of crisis is inevitable [20]. Likewise, having a central reporting system to track and to report causes and extents of lack of medications in a timely manner to guarantee better responses in times of drug shortages in crisis is essential [21]. The significance of medical supply and preparation in health system’s disaster management is to the extent that “medical products, vaccines and technology” are considered as the priorities in all kinds of hazards [22] and should be mentioned before and during the crisis.

In the next subtheme of inter-sector challenges related to the health system, we can consider challenges in crisis scene management. Human resources are the main issue to consider in this regard. Proper planning and employee training for crisis are thus two key elements that can prepare healthcare systems to act in response to crisis-induced problems [23]. Evidence also implies that weakness in human resources skills and insufficient training when confronting crises and natural disasters are among the obstacles and challenges facing healthcare systems. To enhance these skills, educational topics can be taught to medical students as well as those providing first-line services [24, 25]. In should not be forgotten that these training programs and skill enablers have to be chosen according to the nature of the disaster. For instance, according to Raven et al. (2018), the health workers` lack of knowledge, fear from the disaster particularly in contagious outbreaks and the misconceptions and anxiety can worsen the situation and should be mentioned by the policy makers [26]. Along with these strategies, empowering the local volunteers can be another solution for such these emergency situations particularly for not-specialized services.

The third subtheme of inter-sector challenges related to the health system was service delivery. In this regard, the present results have shown that one of the major challenges was lack of preparedness and proper planning by organizations involved in earthquakes. The shortage of strategic drugs and anesthetics, especially under the influence of economic sanctions, was another challenge recognized in this study. McLean and Whang (2019) have mentioned that economic sanctions can have a negative impact on the country under sanction’s overall budget and the budget allocating to the preparedness for disasters [27].

Other results of the study have shown that, structural challenges are among the intra-sectoral challenges related to the health system. Coordination between governmental and non-governmental organizations was one of the key features of crisis management structure in this category, as there is strong evidence that cooperating with stakeholders and sharing resources such as equipment as well as inter-governmental and non-governmental experiences can improve performance, cost effectiveness, and timely service delivery [28]. Planning and preparedness are also very important factors related to the structural challenges. In this regard, a study in Italy had reported that over 62% of crisis responses had been unplanned and without any preparations and they had been merely practiced at the scene of the events [29].

According to the other results of the study, non-related health system challenges the same as social, cultural and psychological factors are considered among the other challenges of Iranian health system during disasters. First of all, evidence shows that social vulnerability during disasters as a complicated and multidisciplinary concept, can be influenced by many factors the same as the aging structure of the community, the economic status, individual characteristics and cultural variables [30]. In this regard, paying attention to vulnerable groups in times of crisis is vital. Studies have further reported that most disaster-affected groups in crises are at risk [31]. Past crisis experiences also show that no attention to these groups, for example, those in need of psychiatric medications can lead to numerous problems, including destructive behaviors among the injured people and those residing in shelters [32], so having complete information and databases of these patients as well as family members’ knowledge of the types of medications and their symptoms can be very helpful during crises [33].

Another challenges raised in this category was the issue of trust-building and social capital. Evidence shows that crisis management focuses more on infrastructure and equipment such as medical facilities and vaccines, and less on social infrastructure such as trust, education, crisis knowledge, and social capital [34]. Since crisis management is a multifaceted and complex issue, it is necessary to address the social issues of the crisis and to engage the public in it. Mental preparedness among individuals involved in crises can thus reduce the consequences of natural disasters.

In sum, although different studies try to introduce their frame works for disaster management, it seems that in spite of the similarities of the models the same as considering prevention and preparedness before disasters, response phase during the disasters and recovery, relief and rehabilitation right after the previous phase [35] this topic is greatly depending on the context and social, cultural and other non-health related factors. In this regard it is recommended to pay enough attention to the health systems preaddress along with the community’s enabling and sociocultural facilitators for facing disasters.

## Conclusion

Results of this study revealed that, along with health system oriented challenges that can have the inter-sectoral or intra-sectoral nature, the non-health system oriented challenges the same as social, cultural and psychological factors can be considered as the major challenges of Iran’s healthcare system in the face of crises. This complicated context can shed the light to policy makers that not only attention to the medicine and medical equipment supply chain, manpower preparation and service delivery system can be considered as an emergency, but also careful attention to the structural challenges and crisis-scene management should be planned and considered as a priority. Besides, the policy makers and the local managers should try to plan and act in a contingent situation according to the social and cultural characteristics of the region and the psychological condition and the mental needs of the people.

## Supplementary information

**Additional file 1.** The final codes of the Iranian challenges during natural disasters.

## Data Availability

While identifying/confidential patient data should not be published within the manuscript, the datasets used and/or analyzed during the current study are available from the corresponding author on reasonable request.

## Abbreviations

CRED: Center for Research on the Epidemiology of Disasters
WHO: World Health Organization
