# Supplementary material for: Iranian’s healthcare system challenges during natural disasters: the qualitative case study of Kermanshah earthquake
Source: BMC Emerg Med. 2020 Sep 24;20:75. doi: 10.1186/s12873-020-00359-2 (PMC7517634; doi:10.1186/s12873-020-00359-2)
Supplement: Supplementary file 1 — Additional file 1. The final codes of the Iranian challenges during natural disasters. [file 12873_2020_359_MOESM1_ESM.docx]

**Table 2- The final codes of the Iranian challenges during natural disasters**

| **Final codes** |
| --- |
| Local needs` identification according to diversity and population |
| Prioritizing pharmaceutical consumption according to their shelf life |
| Identifying high and low needed pharmaceuticals during disasters |
| Lack of high tech and strategic medicines and medical equipment |
| Depot of imported equipment in the hospitals` stockrooms |
| Lack of strategic storage of pharmaceuticals and medical equipment |
| Lack of strategic medicines depot for encountering disasters |
| Presence of medical equipment companies in provinces supply chain |
| Challenges related to pharmaceutical distribution according to the included area |
| Rebuilding of the hospitals` stocks for preparedness to disasters |
| importance of vaccines` procurement for infants |
| Lack of appropriateness between the increase in the price of initial materials and produced medicines |
| Restriction of medicine production because of increase in the price of initial materials |
| Shortage of domestic producers and manufacturers |
| Lack of domestic production of some medical equipment |
| Dependency of domestic production to imported materials |
| Defining the Structure of disaster management |
| Defining health care management in the disaster structure |
| Lack of national systematic disaster management |
| Coordination between effective organizations before disasters |
| Coordination between public and private sector during disaster |
| Contracting with medical equipment distributors |
| Necessity of plans and budget at the disaster time |
| Art of planning by the managers at the political-economic sanctions |
| Necessity of long term national planning |
| Challenges related to the payments to medical equipment companies |
| Effect of economic situation on the populations` expectation |
| Immediate financing at the time of disaster |
| Shortage of medical equipment and beds for service delivery |
| optimal use of medical equipment at the time of disaster |
| Oldness of medical equipment |
| Dependency of all providers to food and drug department of the medical university |
| Establishment of non-local personnel in the disaster area |
| Request for sending non-local personnel to the disaster area |
| Necessity of the release of local personnel faced with disaster |
| Attention to the safety of the personnel |
| Lack of databases and information banks for medical equipment inventory |
| Necessity of channels for information fluid |
| Creation of information system at the disaster time |
| Applying free capability of hospitals |
| Hospital engagement in the farer areas |
| Destruction of therapeutic infrastructures |
| Building portable hospitals |
| prevention of applying damaged ambulances |
| Necessity of centralized management of ambulances |
| Air plane emergency reinforcement |
| Immediate pre hospital assessment |
| Necessity of quarantine and triage for injured people |
| Mother and child hygiene pack preparation |
| Disease burden estimation |
| Communicable disease control in disasters |
| Vaccination |
| Lack of people`s trust to the government |
| Lack of people`s trust to the media |
| Lack of people`s trust because of fraud and un achievement of previous promises |
| Identification of effective factors on people1s trust to the governmental organizations during disasters |
| Cultural dimension as a macro factor in disasters |
| Effect of cultural social situation on the pharmaceutical and medical equipment supply chain |
| The sense of pity towards victims and injured |
| Identification of vulnerable groups (children, old people, mental disordered ) |
| Familiarity with the importance and dimensions of mental health in disasters |
| The importance of people`s last experiences in disasters |
